# Homologs of genes expressed in *Caenorhabditis elegans *GABAergic neurons are also found in the developing mouse forebrain

**DOI:** 10.1186/1749-8104-5-32

**Published:** 2010-12-01

**Authors:** Elizabeth AD Hammock, Kathie L Eagleson, Susan Barlow, Laurie R Earls, David M Miller, Pat Levitt

**Affiliations:** 1 Department of Pediatrics, Vanderbilt University School of Medicine, Nashville, TN 37232, USA; 2 Vanderbilt Kennedy Center, Vanderbilt University, Nashville, TN 37232, USA; 3 Zilkha Neurogenetic Institute, Keck School of Medicine, University of Southern California, Los Angeles, CA 90089, USA; 4 Department of Cell and Developmental Biology, Vanderbilt University, Nashville, TN 37232-8240, USA; 5 Program in Neuroscience, Vanderbilt University, Nashville, TN 37232-8240, USA; 6 Siskin Hospital for Physical Rehabilitation, One Siskin Plaza, Chattanooga, TN 37403, USA; 7 St Jude Children's Research Hospital, Memphis, TN 38105, USA

## Abstract

**Background:**

In an effort to identify genes that specify the mammalian forebrain, we used a comparative approach to identify mouse homologs of transcription factors expressed in developing *Caenorhabditis elegans *GABAergic neurons. A cell-specific microarray profiling study revealed a set of transcription factors that are highly expressed in embryonic *C. elegans *GABAergic neurons.

**Results:**

Bioinformatic analyses identified mouse protein homologs of these selected transcripts and their expression pattern was mapped in the mouse embryonic forebrain by *in situ *hybridization. A review of human homologs indicates several of these genes are potential candidates in neurodevelopmental disorders.

**Conclusions:**

Our comparative approach has revealed several novel candidates that may serve as future targets for studies of mammalian forebrain development.

## Background

Proper forebrain patterning and cell-fate specification lay the foundation for complex behaviors. These neurodevelopmental events in large part depend on a series of gene expression refinements (reviewed in [1]) that commit cells to express certain phenotypic features that define circuit formation. Relatively subtle disturbances in development may underlie the etiology of neurodevelopmental disorders, especially when alternative cognitive phenotypes do not have an apparent malformation at the gross anatomical level. In the forebrain, cells producing γ-aminobutyric acid (GABAergic interneurons) have been implicated in neurodevelopmental disorders, including autism and schizophrenia [2-4]. These neurons are composed of a diverse class of cells providing a wide range of control of neural activity, and vary in neuroanatomical location, electrophysiological properties, transcriptome/proteome and innervation patterns as either local circuit or long-range projection neurons [5]. As with other cell types, the diversity of GABAergic neurons has its basis in different developmental origins, with timing and location of birth playing key roles in cell fate [1,6-8].

Despite the phenotypic variety of GABAergic neurons, all use GABA as a neurotransmitter. In mammals, GABA is produced by one of two GABA-synthesizing enzymes, glutamic acid decarboxylase (GAD)65 or GAD67. These closely related enzymes are orthologs of the *Caenorhabditis elegans *protein UNC-25, which is found only in cells that produce GABA. Because UNC-25/GAD and other components of the GABA synthetic pathway are highly conserved, it is likely that mammalian orthologs of some of the genes that specify GABAergic cell fate in *C. elegans *embryogenesis may also control GABAergic fate specification during mammalian embryogenesis.

**Figure 1:**
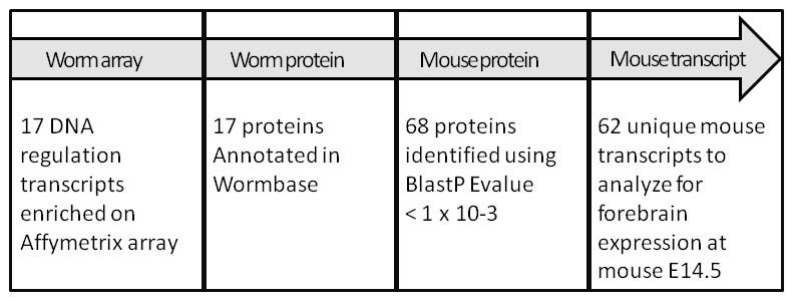
**Summary diagram of experimental approach**.

We have explored this hypothesis in an effort to define new candidates for regulating forebrain GABAergic cell fate that may be highly conserved across evolutionarily distant taxa. This discovery-based approach (Figure 1) complements existing analyses of the transcriptomes of subpopulations of mammalian GABAergic cells [9-13]. Thus, by using data from the transcription profiling of GABAergic cells in embryonic *C. elegans*, in combination with bioinformatics analyses, we report here transcripts with sequence homologs that may also be involved in GABAergic fate specification in mammals. We focused our attention on transcripts with gene regulation ontologies. To probe the potential role of these conserved players in mammalian development, we mapped these gene products in the developing mouse forebrain, with a selective focus on the telencephalon. As a proof of principle, this strategy identified several gene products already known to play a role in the specification of forebrain GABAergic interneurons in mammals. Additionally, our approach identified several previously unexplored gene products that serve as promising candidates for future investigation of forebrain patterning.

## Materials and methods

### *C. elegans *transcription profiling

A microarray profiling of *C. elegans *cells (MAPCeL) strategy was used to obtain a transcriptome profile of *C. elegans *GABAergic neurons [14,15]. A complete description of the methods used for this study and the GABAergic neuron expression profile will be reported elsewhere (S Barlow, L Earls, J Watson, C Spencer, K Watkins, D Miller, manuscript in preparation). Briefly, the *unc-25::GFP *marker was used to label *C. elegans *GABAergic neurons. *unc-25::GFP*-expressing embryos were dissociated with chitinase and cultured for 24 hours and viable *unc-25::GFP *labeled cells were isolated by fluorescence activated cell sorting (FACS). Total RNA was purified from both the sorted *unc-25::GFP *positive cells and from the reference sample of all embryonic cells. The RNAs were amplified and hybridized to the Affymetrix *C. elegans *array. Average signal intensities were calculated from three independent isolates of the *unc-25::GFP *cells and from four replicates of the reference samples. A comparison of the *unc-25::GFP *and reference data sets identified 673 transcripts showing elevated expression (1.7×) in GABAergic neurons at a false discovery rate (FDR) ≤ 1% [14]).

### Bioinformatics screen

Genes in the list of GABAergic enriched transcripts with Gene Ontology (GO) terms related to DNA and transcription regulation were analyzed for potential homology to mouse transcripts. Because functional homology is conserved at the protein level, we generated a list of *C. elegans *proteins from the list of corresponding transcripts and then used BLASTP [16] analysis available at WormBase [17] from June 2005 to November 2008 (wormbase releases WS144 to WS196) to identify the closest matching mouse protein sequence homologs. We then used this list of mouse protein homologs to generate the corresponding catalogue of mouse transcripts for *in situ *hybridization analysis. We did not distinguish among potential splice variants and/or protein isoforms for a given single gene locus. To further rank potential candidates, we performed BLASTP in the reverse direction; after generating the list of mouse protein sequence homologs, those proteins were used to identify the best sequence homologs in the *C. elegans *proteome.

### Mouse care and use

Timed pregnant C57Bl6j mice were bred in-house from founders originating from Jackson Labs under protocols approved by the Institutional Animal Care and Use Committee of Vanderbilt University. Mice were maintained on a 12:12 light-dark cycle and were permitted food and water *ad libitum*. Noon on the day following a time-delimited overnight pairing was considered embryonic day 0.5 (E0.5). Pregnant females were readily identifiable at E14.5 and were deeply anesthetized with isofluorane vapors followed by rapid decapitation in order to harvest embryos. Expression patterns of genes at this fetal age were analyzed because it is a mid-point in the age-range for cortical GABAergic neuron production and migration in the mouse forebrain [8]. Thus, we hypothesized that expression patterns related to GABAergic neuron specification and differentiation likely would be apparent at this age.

### Riboprobe labeling

I.M.A.G.E. clones were obtained from ATCC (Manassas, VA, USA) and Open Biosystems (Huntsville, AL, USA) for the mouse transcripts (Additional file 1). The identity of each I.M.A.G.E. clone was confirmed by sequencing at the Vanderbilt DNA Sequencing Facility. When necessary, due to cDNA size or the plasmid vector, we subcloned the I.M.A.G.E. clone into a separate vector (Additional file 2). These subclones were also sequenced to confirm identity and orientation. Plasmids were linearized and transcribed using T7, Sp6 or T3 polymerase (Promega, Madison, WI, USA) depending on the plasmid vector, by standard methods. Digoxigenin-11-uridine-5'-triphosphate (0.35 mM; Roche, Indianapolis, IN, USA) was included in the transcription reaction to allow for non-radioactive colorimetric detection of transcripts.

### *In situ *hybridization

Fetuses at E14.5 were harvested into cold phosphate-buffered saline and crown-rump length (11 to 12 mm) confirmed. Whole heads or microdissected brains were immersion fixed for 24 hours in 4% formaldehyde in 0.156 M NaH_2_PO_4_, 0.107 M NaOH, pH 7.12 with HCl. After fixation, brains were cryoprotected in graded 10, 20 and 30% sucrose in phosphate-buffered saline followed by embedding in TFM Tissue Freezing Medium (Triangle Biomedical Sciences, Inc., Durham, NC, USA) over liquid nitrogen. Brains were stored at -80°C until cryostat sectioning into 6 series at 20 microns each. Slides containing the tissue were stored at -80°C until they were fixed, acetylated and dehydrated, and then returned to -80°C until *in situ *hybridization was performed. *In situ *hybridization was performed on a Tecan Evo 150 (Tecan Group Ltd, Männendorf, Switzerland) following the Allen Brain Atlas [18] and GenePaint [19] protocols (Additional files 3 and 4). After the machine completed the described protocol, BCIP and NBT (Roche) were applied manually. The time in color development ranged from 30 minutes to 4 hours. After color development, the slides were rinsed four times with double distilled water and then twice with 4% formaldehyde. Slides were removed from the machine, dehydrated through a series of alcohols and coverslipped with VectaMount (Vector Laboratories, Burlingame, CA, USA).

### Light microscopy

Microscopy was performed using an Axioplan II microscope (Zeiss, Jena, Germany), and micrographs were acquired with a Zeiss AxioCam HRc camera (Zeiss) in Axiovision 4.1 software (Zeiss). Low-magnification images were collected and linearly adjusted for brightness and contrast using Adobe Photoshop (version 7.0, Adobe, San Jose, CA, USA). No other image alterations other than resizing were performed. All figures were prepared digitally in PowerPoint 2007 (Microsoft, Redmond, WA, USA).

## Results

### Genes expressed in *C. elegans *GABAergic neurons

*C. elegans *embryonic GABAergic neurons were profiled by the MAPCeL approach in which *unc-25::GFP *labeled cells were isolated by FACS for microarray analysis. Comparison to a reference data set obtained from all embryonic cells revealed 673 transcripts with enriched (1.7×) expression in GABAergic neurons. Strong enrichment of established GABAergic neuron markers, such as *unc-25 *(glutamic acid decarboxylase; 61×), *unc-47 *(vesicular GABA transporter; 7×) and *acr-9 *(nicotinic acetylcholine receptor; 25×) [20,21] indicate that other transcripts in this data set are also likely to be highly expressed in embryonic *C. elegans *GABAergic neurons *in vivo *(S Barlow, L Earls, J Watson, C Spencer, K Watkins, D Miller, manuscript in preparation). Seventy five percent of the highly expressed transcripts had defined gene ontologies and of those, 17 transcripts (2.5%) in this list met criteria for DNA regulation-related gene ontologies (Table 1).

**Table 1:** Transcription regulation genes with enriched expression in embryonic C. elegans GABAergic cells

Worm transcript	Fold change	KOG
unc-30	20.11	Transcription factor PTX1, contains HOX domain
fkh-10	9.64	Transcription factor of the Forkhead/HNF3 family
bar-1	4.94	Armadillo/beta-Catenin/plakoglobin
F30A10.3	3.28	Inositol polyphosphate multikinase, component of the ARGR transcription regulatory complex
alr-1	3.23	Transcription factor, contains HOX domain
ceh-27	3.03	Transcription factor tinman/NKX2-3, contains HOX domain
nhr-47	2.74	Hormone receptors
nhr-190	2.48	Hormone receptors
F53H10.2	2.36	Predicted DNA-binding protein, contains SANT and ELM2 domains
mes-2	2.20	Transcriptional repressor EZH1
taf-11.1	1.93	Transcription initiation factor TFIID, subunit TAF11
nhr-4	1.85	Hormone receptors
ceh-44	1.84	Transcription factor/CCAAT displacement protein CDP1
mdt-6	1.82	RNA polymerase II transcriptional regulation mediator
aly-2	1.79	RRM motif-containing protein
mdt-8	1.74	Uncharacterized conserved protein
hlh-11	1.73	bHLH transcription factor

### Bioinformatics assessment of mouse homologs

The original list of 17 *C. elegans *candidate transcription factors was used to identify 68 mouse homologs by BLASTP with an expectation cut off of ≤ E-3 (Table 2). The average number of mouse homologs was 3.8 for each *C. elegans *protein, with a mode of 3, a minimum of 2 and a maximum of 8 sequence homologs. Because of the similarity among certain *C. elegans *transcripts, three mouse proteins (Hnf4A, Hnf4G and Ezh2) appeared on the list more than once. When considering these duplications, there were 62 unique gene products to pursue for expression analysis. This analytical strategy appears to be suitable for identifying neurodevelopmental candidates, as we found that several mouse orthologs with homology to *C. elegans *transcripts have a known role in forebrain patterning. In particular, genes with selective roles in determining GABAergic phenotype in mammals were identified, including known players in the forebrain (*Nkx2. 1 *[22], *Arx *[23], *Cux2 *[24]), midbrain (*Pitx2 *[25]) and spinal cord (*Cux2 *[26]).

**Table 2:** Mouse homologs by protein sequence homology

Worm transcript	Mouse gene	Gene synonyms	Description	E value	R BLASTP
unc-30	*Pitx1*	*Bft*, *Potx*, *Ptx1*	Pituitary homeobox 1 (Paired-like homeodomain transcription factor 1) (Homeobox protein P-OTX) (Pituitary OTX-related factor) (Hindlimb-expressed homeobox protein backfoot)	3.9E-28	Yes
	*Pitx2*	*Arp1*, *Brx1*, *Otlx2*, *Ptx2*, *Rgs*	Pituitary homeobox 2 (Paired-like homeodomain transcription factor 2) (Homeobox protein PITX2) (Orthodenticle-like homeobox 2) (Solurshin) (ALL1-responsive protein ARP1) (BRX1 homeoprotein) (Paired-like homeodomain transcription factor Munc 30)	4.8E-28	Yes
	*Pitx3*		Pituitary homeobox 3 (Paired-like homeodomain transcription factor 3) (Homeobox protein PITX3)	1.2E-26	Yes
fkh-10	*Foxb2*	*Fkh4*	Forkhead box protein B2 (Transcription factor FKH-4)	2.6E-27	No
	*FoxL1*	*Fkh6*, *Fkhl11*	Forkhead box protein L1 (Forkhead-related protein FKHL11) (Transcription factor FKH-6)	4.2E-27	No
	*Foxa1*	*Hnf3a*, *Tcf-3a*, *Tcf3a*	Hepatocyte nuclear factor 3-alpha (HNF-3A) (Forkhead box protein A1)	6.2E-27	No
	*Foxb1*	*Fkh5*, *Foxb1a*, *Foxb1b*, *Mf3*	Forkhead box protein B1 (Transcription factor FKH-5)	1.1E-26	No
	*Foxi2*		Forkhead box protein I2	1.2E-26	No
	*Foxd2*	*Mf2*	Forkhead box protein D2 (Mesoderm/mesenchyme forkhead 2)	1.8E-26	No
	*Foxq1*	*Hfh1*, *Hfh1l*	Forkhead box protein Q1 (Hepatocyte nuclear factor 3 forkhead homolog 1) (HNF-3/forkhead-like protein 1) (HFH-1l)	6.5E-26	No
	*Foxa3*	*Hnf3g*, *Tcf-3g*, *Tcf3g*	Hepatocyte nuclear factor 3-gamma (Forkhead box protein A3)	7.4E-26	No
bar-1	*Jup*		Junction plakoglobin (Desmoplakin-3) (Desmoplakin III)	5.2E-43	No
	*Ctnnb1*	*Catnb*	Catenin beta-1 (Beta-catenin)	1.1E-42	No
F30A10.3	*Ip6k1*	*Ihpk1*	Inositol hexakisphosphate kinase 1 (Inositol hexaphosphate kinase 1)	3.7E-54	Yes
	*Ip6k2*	*Ihpk2*	Inositol hexakisphosphate kinase 2 (P(i)-uptake stimulator/PiUS)	1.6E-48	Yes
	*Ip6k3*	*Ihpk3*	Inositol hexakisphosphate kinase 3 (Inositol hexaphosphate kinase 3)	5.9E-48	Yes
	*Ipmk*	*Impk*	Inositol polyphosphate multikinase (Inositol 1,3,4,6-tetrakisphosphate 5-kinase)	1.4E-08	No
alr-1	*Phox2a*	*Arix*, *Phox2*, *Pmx2*, *Pmx2a*	Paired mesoderm homeobox protein 2A (Paired-like homeobox 2A) (PHOX2A homeodomain protein) (Aristaless homeobox protein homolog)	5.6E-28	No
	*Arx*		Homeobox protein ARX (Aristaless-related homeobox)	5.9E-28	Yes
	*Alx4*		Homeobox protein aristaless-like 4 (ALX-4)	5.1E-26	Yes
	*Phox2b*	*Pmx2b*	Paired mesoderm homeobox protein 2B (Paired-like homeobox 2B) (PHOX2B homeodomain protein) (Neuroblastoma Phox/NBPhox)	3.6E-25	No
	*Pax7*	*Pax-7*	Paired box protein Pax-7	6.7E-25	No
ceh-27	*Nkx2-5*	*Csx*, *Nkx-2.5*, *Nkx2e*	Homeobox protein Nkx-2.5 (Homeobox protein NK-2 homolog E) (Cardiac-specific homeobox) (Homeobox protein CSX)	3.6E-22	No
	*Nkx2-3*	*Nkx-2.3*, *Nkx2c*	Homeobox protein Nkx-2.3 (Homeobox protein NK-2 homolog C) (Nkx2-C) (Homeobox protein NK-2 homolog 3)	6.0E-20	No
	*Nkx2-1*	*Nkx-2.1*, *Titf1*, *Ttf1*	Homeobox protein Nkx-2.1 (Thyroid transcription factor 1/TTF-1) (Thyroid nuclear factor 1)	2.1E-18	No
	*Nkx2-4*	*Nkx2d*	Homeobox protein Nkx-2.4 (Homeobox protein NK-2 homolog D)	2.2E-18	No
nhr-47	*Hnf4g*	*Nr2a2*	Hepatocyte nuclear factor 4-gamma/HNF-4-gamma (Nuclear receptor subfamily 2 group A member 2)	7.7E-35	No
	*Hnf4a*	*Hnf-4*, *Hnf4*, *Nr2a1*, *Tcf14*	Hepatocyte nuclear factor 4-alpha/HNF-4-alpha (Transcription factor HNF-4) (Nuclear receptor subfamily 2 group A member 1) (Transcription factor 14)	7.4E-34	No
nhr-190	*Hnf4a*	*Hnf-4*, *Hnf4*, *Nr2a1*, *Tcf14*	Hepatocyte nuclear factor 4-alpha/HNF-4-alpha (Transcription factor HNF-4) (Nuclear receptor subfamily 2 group A member 1) (Transcription factor 14)	2.3E-12	No
	*Hnf4g*	*Nr2a2*	Hepatocyte nuclear factor 4-gamma/HNF-4-gamma (Nuclear receptor subfamily 2 group A member 2)	7.1E-12	No
	*Rarg*	*Nr1b3*	Retinoic acid receptor gamma/RAR-gamma (Nuclear receptor subfamily 1 group B member 3)	1.1E-09	No
F53H10.2	*Znf541*	*Ship1*, *Zfp541*	Zinc finger protein 541 (Spermatogenic cell HDAC-interacting protein 1)	4.2E-31	Yes
	*Trerf1*		Transcriptional-regulating factor 1 (Transcriptional-regulating protein 132) (Zinc finger transcription factor TReP-132)	1.4E-28	Yes
	*C130039O16Rik*		Putative uncharacterized protein	1.2E-17	Yes
	*Mier1*	*Kiaa1610*	Mesoderm induction early response protein 1/Mi-er1	2.7E-04	No
	*Rcor1*	*D12Wsu95e*, *Kiaa0071*	REST corepressor 1 (Protein CoREST)	2.7E-04	No
	*Foxj3*	*Kiaa1041*	Forkhead box protein J3	4.3E-03	No
	*Ncor1*	*Rxrip13*	Nuclear receptor corepressor 1/N-CoR1/N-CoR (Retinoid X receptor-interacting protein 13/IP13)	6.7E-03	No
mes-2	*Ezh2*	*Enx1h*	Histone-lysine N-methyltransferase EZH2 (Enhancer of zeste homolog 2) (ENX-1)	3.0E-60	Yes
	*Ezh1*	*Enx2*	Histone-lysine N-methyltransferase EZH1 (Enhancer of zeste homolog 1) (ENX-2)	8.2E-56	Yes
	*Suv39h1*	*Suv39h*	Histone-lysine N-methyltransferase SUV39H1 (Suppressor of variegation 3-9 homolog 1) (Position-effect variegation 3-9 homolog) (Histone H3-K9 methyltransferase 1) (H3-K9-HMTase 1)	5.1E-13	No
taf-11.1	*Taf11*		Transcription initiation factor TFIID subunit 11 (Transcription initiation factor TFIID 28 kDa subunit/TAF(II)28/TAFII-28/TAFII28) (TFIID subunit p30-beta)	3.0E-24	Yes
	*Dspp*	*Dmp3*	Dentin sialophosphoprotein precursor (Dentin matrix protein 3/DMP-3) [Cleavage products: Dentin phosphoprotein (Dentin phosphophoryn/DPP); Dentin sialoprotein/DSP]	1.3E-04	No
	*Myst3*	*Moz*	Histone acetyltransferase MYST3 (MOZ, YBF2/SAS3, SAS2 and TIP60 protein 3) (Monocytic leukemia zinc finger protein) (Monocytic leukemia zinc finger homolog)	6.4E-04	No
nhr-4	*Hnf4a*	*Hnf-4*, *Hnf4*, *Nr2a1*, *Tcf14*	Hepatocyte nuclear factor 4-alpha/HNF-4-alpha (Transcription factor HNF-4) (Nuclear receptor subfamily 2 group A member 1) (Transcription factor 14)	1.7E-35	No
	*Hnf4g*	*Nr2a2*	Hepatocyte nuclear factor 4-gamma/HNF-4-gamma (Nuclear receptor subfamily 2 group A member 2)	1.5E-31	No
	*Rxrb*	*Nr2b2*	Retinoic acid receptor RXR-beta (Retinoid X receptor beta) (Nuclear receptor subfamily 2 group B member 2) (MHC class I regulatory element-binding protein H-2RIIBP)	2.8E-27	No
ceh-44	*Cux1*	*Cutl1*, *Cux*, *Kiaa4047*	Homeobox protein cut-like 1 (CCAAT displacement protein/CDP) (Homeobox protein Cux)	8.3E-75	Yes
	*Cux2*	*Cutl2*	Homeobox protein cut-like 2/Cut-like 2 (Homeobox protein Cux-2)	3.5E-61	Yes
	*Cux1*	*Cutl1*	Protein CASP	1.9E-39	Yes
	*Myh8*	*Myhsp*	Myosin-8 (Myosin heavy chain 8) (Myosin heavy chain, skeletal muscle, perinatal/MyHC-perinatal)	9.1E-16	No
	*Myh10*		Myosin-10 (Myosin heavy chain 10) (Myosin heavy chain, non-muscle IIb) (Non-muscle myosin heavy chain IIb) (Cellular myosin heavy chain, type B) (Non-muscle myosin heavy chain B)	1.3E-15	No
	*Clip1*	*Kiaa4046*, *Rsn*	CAP-Gly domain-containing linker protein 1 (Restin)	6.0E-15	No
	*Myh11*		Myosin-11 (Myosin heavy chain 11) (Myosin heavy chain, smooth muscle isoform) (SMMHC)	7.3E-14	No
mdt-6	*Med6*		Mediator of RNA polymerase II transcription subunit 6 (Mediator complex subunit 6)	1.7E-25	Yes
	*Rpgrip1*		X-linked retinitis pigmentosa GTPase regulator-interacting protein 1/RPGR-interacting protein 1	5.6E-04	No
	*Sptbn1*		Spectrin beta chain, brain 1 (Spectrin, non-erythroid beta chain 1) (Beta-II spectrin) (Fodrin beta chain) (Embryonic liver fodrin)	7.5E-03	No
	*Ncl*	*Nuc*	Nucleolin (Protein C23)	8.4E-03	No
	*Pnn*		Pinin	8.5E-03	No
aly-2	*Thoc4*	*Aly*, *Ref1*, *Refbp1*	THO complex subunit 4/Tho4 (Ally of AML-1 and LEF-1) (Transcriptional coactivator Aly/REF) (RNA and export factor-binding protein 1) (REF1-I)	1.3E-20	Yes
	*Refbp2*	*Ref2*	RNA and export factor-binding protein 2	1.6E-14	Yes
	*Fox1*	*A2bp*, *A2bp1*	Fox-1 homolog A (Ataxin-2-binding protein 1)	2.0E-03	No
	*Hist1h1a*	*H1f1*	Histone H1.1 (H1 VAR.3/H1a)	2.0E-03	No
mdt-8	*Ezh2*	*Enx1h*	Histone-lysine N-methyltransferase EZH2 (Enhancer of zeste homolog 2) (ENX-1)	2.4E-61	No
	*Med8*		Mediator of RNA polymerase II transcription subunit 8 (Mediator complex subunit 8) (Activator-recruited cofactor 32 kDa component/ARC32)	2.7E-23	Yes
	*Pou6f2*		POU domain, class 6, transcription factor 2	1.1E-03	No
hlh-11	*Tcfap4*	*Ap4*	Activator protein 4 (Putative uncharacterized protein) (Transcription factor AP4)	1.5E-17	Yes
	*Hey2*	*Chf1*, *Herp*, *Herp1*, *Hesr2*, *Hrt2*	Hairy/enhancer-of-split related with YRPW motif protein 2 (Hairy and enhancer of split-related protein 2/HESR-2) (Hairy-related transcription factor 2/mHRT2) (HES-related repressor protein 2) (Protein gridlock homolog)	3.4E-07	No

Performing the reverse BLASTP from mouse proteins to worm proteins informed the strength of the sequence homology for the mouse and worm proteins relative to the other potential homologues in *C. elegans*. This reverse BLASTP can help rank-order candidates for further functional assessment in the future. If the reverse BLASTP returned the original *C. elegans *as the hit with the highest E-value, then 'yes' was entered in the R BLASTP column in Table 2. If the reverse BLASTP had a different *C. elegans *protein as the top hit, then a value of 'no' was entered in Table 2. Of the 68 mouse proteins, 22 had the original worm protein as the top reciprocal hit for sequence homology in the reverse BLASTP.

### *In situ *hybridization mapping of mouse sequence homologs

**Figure 2:**
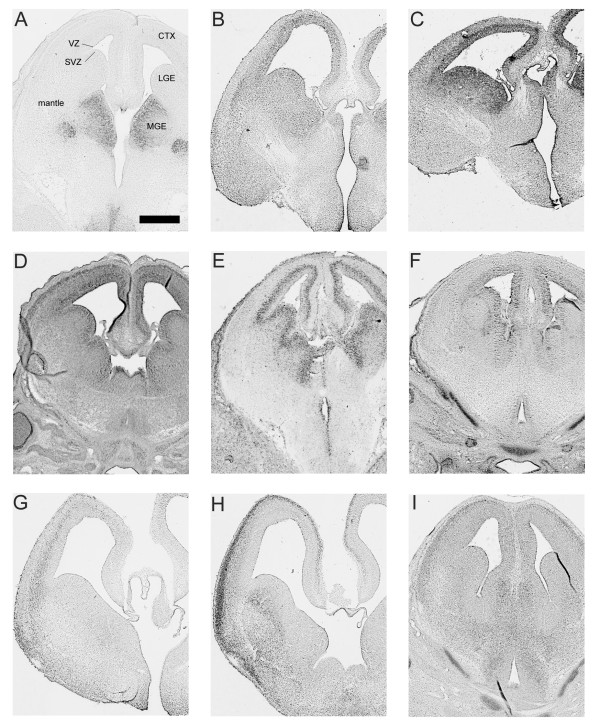
**Examples of the variety of expression patterns at E14.5**.**(A) **Restricted; **(B) **broad; **(C-F) **proliferative zone; **(G-I) **post-mitotic regions. Scale bar = 500 μm. (A) *Nkx2.1*, (B) *Cux1*, (C) *Suv39h1*, (D) *Ezh2*, (E) *Hist1h1a*, (F) *Ncl*, (G) *Cux2*, (H) *Fox1*, (I) *Myst3*. CTX, cortex; LGE, lateral ganglionic eminence; MGE, medial ganglionic eminence; SVZ, subventricular zone; VZ, ventricular zone.

Our criterion for potential relevance of mouse gene products in the specification of telencephalic interneurons was that transcripts must be present in known GABAergic proliferative zones (such as the medial, lateral and caudal subdivisions of the ganglionic eminence), although they need not be exclusively expressed in those brain areas. Representative expression patterns are depicted in Figure 2 with complete results summarized in Table 3. In addition to the expression data generated here, other sources for assessment and/or confirmation of expression were used, including GenePaint [19], Brain Gene Expression Map (BGEM) [27] and the Allen Brain Atlas [18].

**Table 3:** Summary of transcript expression in C57Bl6j mice from E13.5 to E15.5

	Brain expression					VZ					SVZ					Mantle					Cortex				
					
Mouse gene name	V 14.5	G 14.5	A 13.5	A 15.5	B 15	V 14.5	G 14.5	A 13.5	A 15.5	B 15	V 14.5	G 14.5	A 13.5	A 15.5	B 15	V 14.5	G 14.5	A 13.5	A 15.5	B 15	V 14.5	G 14.5	A 13.5	A 15.5	B 15
*Pitx1*	+	+	ND	ND	ND	-	-				-	-				-	-				-	-			
*Pitx2*	+	ND	+	ND	+	-		-		-	-		-		-	-		-		-	-		-		-
*Pitx3*	+	ND	ND	ND	-	-				-	-				-	-				-	-				-
*Foxb2*	-	ND	ND	+	ND	-			-		-			-		-			-		-			-	
*FoxL1*	ND	ND	ND	ND	ND																				
*Foxa1*	-	ND	+	+	+	-		-	-	-	-		-	-	-	-		-	-	-	-		-	-	-
*Foxb1*	-	+	+	+	ND	-	-	-	-		-	-	-	-		-	-	-	-		-	-	-	-	
*Foxi2*	-	ND	ND	ND	ND	-					-					-					-				
*Foxd2*	ND	+	+	ND	ND		-	+				-	+				-	-				-	+		
*Foxq1*	ND	+	ND	+	ND		-		+			-		-			-		-			-		+	
*Foxa3*	-	+	-	-	-	-	+	-	-	-	-	+	-	-	-	-	+	-	-	-	-	+	-	-	-
*Jup*	+	+	ND	ND	ND	-	-				-	-				-	-				-	-			
*Ctnnb1*	+	+	+	ND	ND	+	+	+			+	+	+			-	+	-			-	+	+		
*Ip6k1*	+	+	ND	ND	ND	-	-				-	+				+	+				+	+			
*Ip6k2*	-	+	ND	ND	ND	-	-				-	+				-	+				-	+			
*Ip6k3*	ND	ND	ND	ND	ND																				
*Ipmk*	+	ND	ND	ND	ND	+					+					+					+				
*Phox2a*	ND	+	ND	-	ND		-		-			-		-			-		-			-		-	
*Arx*	-	ND	+	+	ND	-		+	-		-		+	+		-		+	+		-		+	+	
*Alx4*	-	+	ND	+	ND	-	-		-		-	+		-		-	-		-		-	-		-	
*Phox2b*	-	ND	+	+	ND	-		-	-		-		-	-		-		-	-		-		-	-	
*Pax7*	-	ND	ND	+	ND	-			-		-			-		-			-		-			-	
*Nkx2-5*	-	ND	ND	ND	ND	-					-					-					-				
*Nkx2-3*	+	+	ND	ND	ND	+	-				-	-				-	+				-	-			
*Nkx2-1*	+	+	+	+	ND	-	-	-	-		+	+	+	+		-	-	-	-		-	-	-	-	
*Nkx2-4*	ND	ND	ND	ND	ND																				
*Hnf4g*	-	+	-	-	ND	-	-	-	-		-	-	-	-		-	-	-	-		-	-	-	-	
*Hnf4a*	-	ND	ND	ND	ND	-					-					-					-				
*Rarg*	-	+	ND	+	ND	-	+		-		-	-		-		-	-		-		-	+		-	
*Znf541*	ND	ND	ND	ND	ND																				
*Trerf1*	-	+	ND	ND	ND	-	+				-	-				-	-				-	+			
C130039O16Rik	-	+	ND	ND	ND	-	+				-	-				-	-				-	+			
*Mier1*	-	ND	ND	ND	ND	-					-					-					-				
*Rcor1*	-	+	+	+	ND	-	+	+	+		-	-	+	+		-	+	+	-		-	-	+	-	
*Foxj3*	+	ND	ND	+	ND	+			-		+			-		-			-		-			-	
*Ncor1*	ND	ND	+	+	+			+	+	+			+	-	-			-	-	-			+	+	+
*Ezh2*	+	+	ND	ND	ND	+	+				+	-				-	+				-	+			
*Ezh1*	-	ND	ND	ND	ND	-					-					-					-				
*Suv39h1*	+	+	ND	ND	+	+	+			+	+	-			-	-	-			-	+	-			-
*Taf11*	-	+	ND	ND	ND	-	+				-	-				-	-				-	+			
*Dspp*	-	ND	ND	ND	ND	-					-					-					-				
*Myst3*	+	ND	ND	ND	ND	+					-					+					+				
*Rxrb*	+	+	-	-	ND	+	+	-	-		-	-	-	-		-	-	-	-		+	+	-	-	
*Cux1*	+	ND	+	ND	ND	+		+			+		+			+		-			+		+		
*Cux2*	+	+	+	+	ND	-	-	-	-		-	-	-	-		+	+	+	+		+	+	+	+	
*Myh8*	+	ND	ND	ND	ND	+					-					-					-				
*Myh10*	ND	+	ND	ND	ND		+					-					+					+			
*Clip1*	-	+	ND	ND	ND	-	+				-	-				-	+				-	+			
*Myh11*	ND	ND	ND	ND	ND																				
*Med6*	-	+	-	-	ND	-	+	-	-		-	-	-	-		-	-	-	-		-	-	-	-	
*Rpgrip1*	ND	+	ND	ND	-		+			-		-			-		-			-		+			-
*Sptbn1*	-	+	ND	ND	ND	-	+				-	-				-	-				-	+			
*Ncl*	+	+	ND	ND	+	+	+			+	-	-			-	-	-			-	-	-			+
*Pnn*	+	ND	+	ND	+	+		+		+	-		-		-	-		-		-	-		-		-
*Thoc4*	+	+	ND	ND	ND	+	+				+	-				-	-				-	-			
*Refbp2*	+	ND	ND	ND	ND	+					-					-					+				
*Fox1*	+	+	ND	ND	ND	-	-				-	-				+	+				+	+			
*Hist1h1a*	+	ND	ND	ND	ND	+					+					+					+				
*Med8*	-	+	ND	ND	-	-	+			-	-	-			-	-	-			-	-	+			-
*Pou6f2*	+	+	+	ND	ND	-	-	-			-	-	-			-	+	-			-	-	-		
*Tcfap4*	+	+	+	-	ND	+	+	+	-		+	-	+	-		+	-	-	-		+	+	+	-	
*Hey2*	ND	+	+	+	ND		-	+	-			-	-	-			-	-				+	-	-	

V_14.5 _(Vanderbilt Study), G_14.5 _(GenePaint data), A_13.5 _to A_15.5 _(Allen Brain Atlas data at two ages), B_15 _(BGEM dataset). ND or empty cell = no data. SVZ, subventricular zone; VZ, ventricular zone.

Of the 62 unique transcripts, 57 have sufficient data to ascertain brain expression (Table 3). Of these, 52 (91%) exhibited brain expression. We narrowed our focus to known areas of cortical interneuron generation, migration and maturation, particularly the ganglionic eminences. In particular, we closely examined the proliferative ventricular zone (VZ), subventricular zone (SVZ), mantle of the subpallium and the pallium. A majority (38 of 52, 73%) of transcripts from our list were detected in the VZ, although this expression was not restricted to ventral proliferative zones. Rather, these transcripts were more broadly expressed throughout the dorsal and ventral VZ. Sixty percent (31 of 52) of transcripts were expressed in the cortex, 35% (18 of 52) in the mantle and 33% (17 of 52) in the SVZ. Expression patterns that included multiple embryonic histogenic forebrain areas were evident for the majority of transcripts.

We observed three general patterns of expression (Table 3 and Figure 2): pattern 1, expression throughout the forebrain (for example, *Ctnnb1*, *Tcfap4*); pattern 2, expression in post-mitotic cells based on location in the mantle zone and cortical plate (for example, *Cux2*, *Fox1*, *Myst3*); and pattern 3, expression mainly in proliferative zones (for example, *Hist1h1a*, *Ncl*, *Ezh2*, *Suv39h1*). For patterns 2 and 3, expression was generally mosaic and limited to subsets of cells. Although more rare, we did observe expression of some transcripts in discrete areas, such as the well known pattern of *Nkx2.1 *in the medial ganglionic eminence (MGE; Figure 2) and *Pitx2 *(data not shown) in discrete nuclei outside of established forebrain GABAergic proliferative zones.

### OMIM and disease linkage meta-analysis

**Figure 3:**
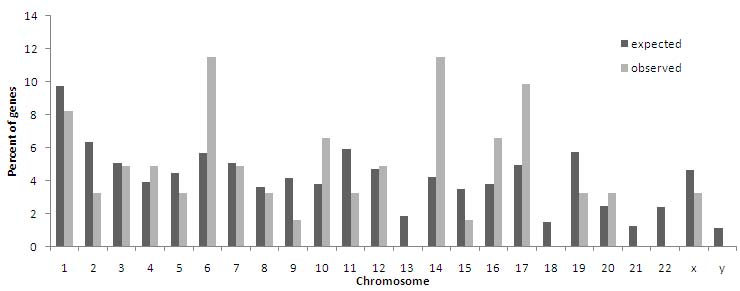
**Distribution of study genes throughout the human genome**.

Human orthologs of the mouse genes were identified through NCBI Homologene. Only one mouse gene, *Refbp2*, does not yet have an identified human ortholog. Manual pBLAST of non-redundant protein entries also revealed no significant human homology to mouse *Refbp2*. The genes identified in this work are scattered throughout the human genome (Figure 3; Additional file 5). In order to assess any potential bias in the distribution of the homologs, we tallied the genes on each chromosome as a percentage of the genes in this study. We then compared those fractions with the distribution of all the genes in the genome (data were obtained from NCBI *Homo Sapiens *build 37.1). A difference score of observed-expected was calculated for each chromosome. We then standardized the difference scores and estimated confidence intervals (degrees of freedom 23). In general, the human homologs of transcripts enriched in worm embryonic GABAergic cells were distributed evenly throughout the genome. The only exception was chromosome 14, in which the standardized difference score fell outside of the 98% confidence interval. Chromosome 6 was just inside the 95% confidence interval, although several of the genes (*IP6K3*, *TAF11*, *TRERF1*, *RXRB*, *HIST1H1A*) cluster near 6p21, a known site of suppressed recombination [28]. This region is associated with reading disability [29] and schizophrenia [30].

To identify known diseases or disorders associated with the identified genes from the *C. elegans *screen, each human gene was used as a search term in Online Mendelian Inheritance in Man (OMIM). Of the 62 transcripts, 17 had OMIM entries. Of these, only three were relevant to neurocognitive phenotypes (Table 4). Mutations in *ARX *are causal for X-linked mental retardation [31], *PHOX2B *mutations are associated with congenital central hypoventilation syndrome [32], and mutations in *NKX2.1 *are associated with congenital chorea [33].

**Table 4:** Transcripts with brain expression were surveyed for evidence of gene association with neurocognitive disorders

Mouse gene	Human gene	Gene association with neurocognitive disorders	Chromosomal position	**Autism endophenotype**[37]
*Pax7*	*PAX7*		1p36.2-p36.12	Language, communication
*Ncl*	*NCL*		2q12-qter	Language, communication
*Ctnnb1*	*CTNNB1*	SZ [51] (protein level)	3p22-p21.3	
*Phox2b*	*PHOX2B*	SZ [52], OMIM 603851	4p12	
*Pitx1*	*PITX1*	ASD [53]	5q31	
*Ezh2*	*EZH2*		7q35-q36	Language, communication; developmental regression
*Ipmk*	*IPMK*		10q21	Social responsiveness
*Nkx2-3*	*NKX2-3*		10q24.2	Social responsiveness
*Pitx3*	*PITX3*		10q25	Social responsiveness
*Foxi2*	*FOXI2*		10q26	Social responsiveness
*Cux2*	*CUX2*	BPD [54]	12q24.12	
*Nkx2-1*	*NKX2-1*	MR [55], OMIM 600635	14q13	
*Tcfap4*	*TFAP4*		16p13	Repetitive behavior/OCD; language, communication
*Fox1*	*A2BP1*	ASD [35], MR and seizures [36]	16p13.3	Repetitive behavior/OCD
*Jup*	*JUP*		17q21	Social responsiveness
*Ezh1*	*EZH1*		17q21.1-q21.3	Social responsiveness
*Hnf4a*	*HNF4A*		20q12-q13.1	Language, communication
*Arx*	*ARX*	ASD, MR, seizures [34], OMIM 300382	Xp22.13	

Autism endophenotype data are reviewed in [37]. ASD, autism spectrum disorder; BPD, bipolar disorder; MR, mental retardation; OCD, obsessive-compulsive disorder; OMIM, Online Mendelian Inheritance in Man; SZ, schizophrenia.

In addition to OMIM analysis, we surveyed the literature for gene association studies that may implicate any of the genes identified in this study with neurocognitive disruption as evident in autism spectrum disorders (ASDs), mental retardation, schizophrenia, seizure disorders or bipolar disorder. These findings are presented in Table 4. *ARX *(reviewed in [34]) is the best-known contributor to phenotypic disturbances among the transcription factors in our list. *A2BP1 *(human *FOX1*) appears to have a similar level of pleiotropy. While *A2BP1 *is relatively understudied, it has been associated with ASD [35], mental retardation and seizure activity [36].

Finally, the hypothesis that disturbances in GABAergic interneurons may play a role in ASD, combined with the emerging interest in endophenotype analysis in trait genetics in ASD, prompted a comparison of the 62 genes to chromosomal regions associated with ASD endophenotypes, rather than association with full ASD diagnosis. Specifically, we relied on summarized evidence from the literature of chromosomal association with autism endophenotype data reviewed by Losh *et al*. [37]. The chromosomal positions of selected genes are presented in Table 4 along with the associated autism endophenotypes for those chromosomal positions. There are several potential candidates for further analysis of autism endophenotypes. In particular, *EZH2 *stands out, as it is located at 7q35-36, within a replicated linkage peak for ASD genetics, including language, communication and developmental regression endophenotypes [38-40]. Additionally, *A2BP1*(*FOX1*) is included in a chromosomal position associated with autism [35,36].

## Discussion

In this report, we adopted a conservation-based bioinformatic approach to identify potential molecular regulators of GABAergic identity in the mammalian telencephalon. GFP-marked GABAergic neurons from the nematode, *C. elegans*, were isolated by FACS for microarray profiling. These data revealed enrichment (≥ 1.7×) of 17 transcripts encoding conserved proteins with potential roles in gene regulation in the nematode. BLASTP of these *C. elegans *proteins identified mouse homologs and 62 independent transcripts corresponding to these mammalian transcription factors were assessed for expression in E14.5 mouse brain. The data generated in our comparative strategy revealed several highly conserved players in GABAergic interneuron differentiation, including *Arx*, *Nkx2.1 *and *Cux2 *[22-24]. The positive identification of these transcripts supports the utility of our bioinformatic approach as a productive strategy for identifying conserved determinants of neuronal fate. Of the reciprocal BLASTP top hits, 14 unique transcripts showed relevant *in situ *hybridization patterns for telencephalic GABAergic neurogenesis, with 3 having known roles (*Arx*, *Cux1*, *Cux2*). Indeed, mutations in *ARX *have been associated with human brain function and interneuron pathology as identified in OMIM [41]. The 11 remaining top reciprocal hits with relevant expression patterns serve as novel candidate genes (*Ip6k1*, *Ip6k2*, *Trerf1*, *C130039O16Rik*, *Ezh2*, *Taf11*, *Med6*, *Thoc4*, *Refbp2*, *Med8*, *Tcfap4*). While not top reciprocal hits, based on striking expression pattern alone, *Hist1h1a*, *Fox1*, *Myst3 *and *Suv39h1 *warrant further attention. This is especially true as reciprocity is not a perfect predictor of candidacy, as two proteins with known function in GABAergic specification were not top reciprocal hits (NKX2.1 and beta-Catenin).

Mammalian GABAergic cells are generated in the preoptic area and ganglionic eminence of the ventral pallium during embryogenesis [8,42-44]. The three main subdivisions of the ganglionic eminence-lateral (LGE), medial (MGE) and caudal (CGE)-generate a diverse portfolio of GABAergic cells. The LGE produces GABAergic projection neurons of the striatum and interneurons of the amygdala and the olfactory bulbs whereas the MGE and CGE produce the majority of cortical and striatal interneurons, although each contributes a different repertoire of cell types. Cells from the MGE (for example, *Nkx2.1*-expressing cells) settle in cortical layers in an inside-out fashion based on cell birth date, whereas the most ventral MGE cells generate neurons of the globus pallidus and striatal cholinergic neurons [45]. In contrast, cells from the CGE tend to migrate to upper layers, independent of birthday, and comprise 15 to 30% of all cortical interneurons [46]. It is curious that of all of the transcription factors that we mapped, *Nkx2.1 *was the only one that was limited to one of the three progenitor pools.

It is clear that the gene regulatory transcripts identified in our study, with the exception of *Nkx2.1*, do not delineate these well-known pools of progenitor populations. The absence of tissue specificity could mean that these transcription factors exercise general roles in neuronal differentiation as opposed to functioning as selective determinants of GABAergic fate. However, the broader expression beyond the boundaries of these defined progenitor zones does not preclude a role for the protein products of these transcripts in contributing to the development of a selective neuronal type. For example, these candidates may be permissive for a particular fate or act in combination with other gene products with more limited expression patterns.

The data generated by our comparative approach blend with and add to the existing data on mammalian transcription factors that could play a role in the full development of GABAergic fates. There have been several efforts in mouse embryogenesis to use transcription profiling of microdissected GABAergic proliferative zones or fluorescent sorting of enhanced GFP (EGFP)-positive interneurons in dissected embryonic brain. For example, Batista-Brito *et al*. [9] used FACS to isolate embryonic interneurons from presumptive neocortex of E13.5 and E15.5 Dlx5/6^Cre-IRIS-EGFP ^mice. They contrasted the transcriptomes of EGFP-positive (interneurons) and EGFP-negative cells (all other cell types) and identified several enriched transcripts, including *Arx *and *Cux2*, as in our study. Because of the region dissected, *Nkx2.1 *was not enriched, as its expression wanes as interneurons leave the medial ganglionic eminence. They also identified several other candidate transcription factors, including some with association with neurological disorders. Faux *et al*. [10] performed a similar experiment contrasting the transcriptomes of interneurons in the cortex versus the ganglionic eminence using GAD67-EGFP FACS isolated cells obtained at E13.5 and E15.5. Among other transcription factors, Faux *et al*. also show increased expression of *Cux2*. *Cux2 *was also identified in a similar study by Marsh *et al*. [11]. By changing the contrasted pools of mRNA, the Faux *et al*. study addressed a different question than the Batista-Brito *et al*. study. The purpose of the Faux *et al*. study was to enrich for transcripts that may play a role in the migration of interneurons, while the Batista-Brito *et al*. study addressed the question of what genes are differentially expressed in interneurons versus other cell types in the embryonic cortex. Clearly, the contrasted pool of mRNA makes a difference in what transcripts appear to play a role in aspects of interneuron specification [9], migration [10] and maturation [12]. Indeed, contrasting mRNA pools from CGE, LGE and MGE can provide candidates for specifying interneuron subtype [13].

While the comparative approach used here has identified novel potential candidates in the specification of interneurons, there are limitations. The experimental design would not detect elements of chromatin structure or microRNAs, for example, as mechanisms of transcriptional regulation. Our analysis was limited to transcripts that encode proteins involved in gene regulation; other protein classes (for example, receptor tyrosine kinases, ion channels) could also be involved. Moreover, the results are correlational; the expression patterns of these novel candidates overlap with areas that produce GABAergic cells, but do not show that these transcripts participate in GABA fate. Functional studies will be necessary to determine a role for these potential novel players. Additionally, while the comparative data used in this study are based on protein sequence homologies, the ultimate goal is to identify functional orthologs across species. Because true functional orthology is determined over time with experimental methods outside of the scope of this manuscript, we implore the reader to view these data as a first step on the path to identifying potential functional orthologs in conserved gene regulation networks to specify a GABAergic fate.

While this comparative approach revealed several highly conserved players in GABAergic neurogenesis, including *Nkx2.1*, *Arx *and *Cux2*, we failed to identify some known factors in mammalian forebrain specification, including *Olig-2*, although we did identify other basic helix-loop-helix (bHLH) transcription factors, such as *Tcfap4*. Also noticeably absent from the list were *Lhx6 *(*lim-4 *in *C. elegans*), *Mash1 *and *Dlx1/2*, all of which have been demonstrated to play a role in GABAergic differentiation in the mammalian forebrain. We note that a related LIM homeodomain protein, LIM-6, is required for differentiation and expression of UNC-25/GAD in a subset of *C. elegans *GABAergic neurons [47].

While *unc-30 *is the top candidate with the highest enrichment in GABAergic cells in the worm data set, none of the mammalian homologs (*Pitx1*, *Pitx2*, *Pitx3*) revealed expression in known GABAergic proliferative zones of the forebrain, even though there was expression in other brain areas at E14.5*. Pitx2 *is highly expressed in GABA neuron progenitors in diencephalon/mesencephalon [48], where it is known to drive *Gad67 *expression [25]. This role is also conserved in the *C. elegans *homolog, *unc-30 *[49]. In fact, both mammalian *Pitx2 *and *C. elegans unc-30 *can both be used to activate *Gad67 *transcription *in vitro *and *in vivo *[25]. While *Pitx2 *and *unc-30 *clearly give rise to a GABA phenotype, based on the absence of *Pitx2 *expression in the forebrain, there are other mechanisms that regulate GABA phenotype in the interneurons of the telencephalon. More than one type of transcription factor or combination of transcription factors likely can drive the GABAergic fate. Indeed, GABAergic fate regulation in the worm offers a striking parallel to the mouse: *unc-30 *drives GABAergic fate in ventral cord motor neurons but not in GABAergic motor neurons in the head where the LIM homeodomain *lim-6 *is required; similarly, *Pitx2 *is highly expressed in diencephalon/mesencephalon GABAergic progenitors and drives *Gad67 *expression but is not required for differentiation of forebrain GABAergic interneurons that depend on ARX. Additionally, *alr-1*, the worm homolog of *ARX*, regulates gene expression in worm GABA motor neurons [50].

## Conclusions

Comparative transcription profiling across diverse taxa is a fruitful approach for generating candidate genes for brain development. Our comparative analysis has pointed to several interesting candidates for the specification of GABAergic cells in the mammalian telencephalon during embryogenesis based on their expression in regions known to produce or contain interneurons. While not exclusively expressed in these regions, *Hist1h1a*, *Ezh2*, *A2bp1 *(*Fox1*), *Suv39h1 *and *Myst3 *are all novel candidates for interneuron development. Furthermore, these candidates represent two relatively understudied classes of gene regulatory proteins in the context of interneuron development, including histone interacting proteins (*Hist1h1a*, *Ezh2*, *Suv39h1 *and *Myst3*) and RNA regulators (*Fox1*/*A2bp1*). As novel candidates for interneuron development, these transcripts may also be candidate genes for, or participate in, pathways giving rise to neurodevelopmental disorders such as autism, mental retardation and schizophrenia. Variation in function of these proteins and their interacting partners might also play a role in brain evolution. These hypotheses remain to be explored.

## Abbreviations

ASD: autism spectrum disorder; CGE: caudal ganglionic eminence; E: embryonic day; EGFP: enhanced green fluorescent protein; FACS: fluorescence activated cell sorting; GABA: γ-aminobutyric acid; GAD: glutamic acid decarboxylase; GFP: green fluorescent protein; LGE: lateral ganglionic eminence; MGE: medial ganglionic eminence; MR: mental retardation; OMIM: Online Mendelian Inheritance in Man; SVZ: subventricular zone; VZ: ventricular zone.

## Competing interests

The authors declare that they have no competing interests.

## Authors' contributions

EADH completed the mouse and human informatics, mapped the expression of the mouse transcripts and co-wrote the manuscript. KLE, LE and SB participated in experimental design and edited the manuscript. DM and PL participated in experimental design and writing the manuscript.

## Supplementary Material

Additional file 1 **Table S1**. IMAGE clones used to generate *in situ *hybridization probes in this study. Click here for file

Additional file 2 **Table S2**. Primers used for the generation of subclones from IMAGE clones used to generate *in situ *hybridization probes in this study. Click here for file

Additional file 3 **Table S3**. Modified Allen Brain Atlas *in situ *hybridization protocol and Eurexpress II SOP on Tecan Evo GenePaint System. Click here for file

Additional file 4 **Table S4**. Buffer compositions for *in situ *hybridization protocol. Click here for file

Additional file 5 **Table S5**. Chromosomal position of human homologs of all study genes. Click here for file
