# Supplementary material for: Homologs of genes expressed in Caenorhabditis elegans GABAergic neurons are also found in the developing mouse forebrain
Source: Neural Dev. 2010 Dec 1;5:32. doi: 10.1186/1749-8104-5-32 (PMC3006369; doi:10.1186/1749-8104-5-32)
Supplement: Additional file 1 — Table S1. IMAGE clones used to generate in situ hybridization probes in this study. [file 1749-8104-5-32-S1.DOCX]

Table S1. IMAGE clones used in this study. IMAGE clones were sequenced to confirm the template sequence and orientation. Appropriate restriction enzymes and RNA polymerases were used for probe generation. Asterisks indicate plasmids that were subcloned before using as probes (see Table S2 for subclone information). N.A. = IMAGE clone not acquired.

| **Mouse Name** | **IMAGE clone** | **linearizing enzyme** | **anti-sense promoter** |
| --- | --- | --- | --- |
| Pitx1 | 4192818 | KpnI | T7 |
| Pitx2 | 30606653 | AscI | T3 |
| Pitx3 | 40129767 | Not1 | T3 |
| Foxb2 | 40105285 | XhoI | Sp6 |
| FoxL1 | N.A. |  |  |
| Foxa1 | 5720113* |  |  |
| Foxb1 | 40045942 | KpnI | Sp6 |
| Foxi2 | 30434074* |  |  |
| Foxd2 | N.A. |  |  |
| Foxq1 | N.A. |  |  |
| Foxa3 | 5101155 | SstII | T7 |
| Jup | 4459882 | KpnI | T7 |
| Ctnnb1 | 5709247 | AscI | T3 |
| Ip6k1 | 6827203 | AscI | T3 |
| Ip6k2 | 2609803* |  |  |
| Ip6k3 | N.A. |  |  |
| Ipmk | 6834748 | SalI | T3 |
| Phox2a | N.A. |  |  |
| Arx | 5707995* |  |  |
| Alx4 | 6506755* |  |  |
| Phox2b | 30360139* |  |  |
| Pax7 | 6843799* |  |  |
| Nkx2-5 | 1315616 | SacI | T7 |
| Nkx2-3 | 6807512* |  |  |
| Nkx2-1 | 6416507 | SalI | T3 |
| Nkx2-4 | N.A. |  |  |
| Hnf4g | 40130034 | PmeI | T7 |
| Hnf4a | 4238842 | KpnI | T7 |
| Rarg | 3989967 | StuI | T7 |
| Znf541 | N.A. |  |  |
| Trerf1 | 6412734 | AscI | T3 |
| C130039O16Rik | 552340* |  |  |
| Mier1 | 1395455* |  |  |
| Rcor1 | 3419361* |  |  |
| Foxj3 | 6314981* |  |  |
| Ncor1 | N.A. |  |  |
| Ezh2 | 3586689 | KpnI | T7 |
| Ezh1 | 3600884 | KpnI | T7 |
| Suv39h1 | 5352230 | SstII/AvrII | T7 |
| Taf11 | 3483315 | KpnI | T7 |
| Dspp | 40049020 | NotI | T7 |
| Myst3 | 5360083* |  |  |
| Rxrb | 5352230 | KpnI | T7 |
| Cux1 | 30547458 | SalI | T3 |
| Cux2 | 30532644 | AscI | T3 |
| Myh8 | 1480571* |  |  |
| Myh10 | N.A. |  |  |
| Clip1 | 3986143* |  |  |
| Myh11 | N.A. |  |  |
| Med6 | 3710612 | KpnI | T7 |
| Rpgrip1 | N.A. |  |  |
| Sptbn1 | 6758850* |  |  |
| Ncl | 3495665* |  |  |
| Pnn | 1532266* |  |  |
| Thoc4 | 40129511 | NotI | T3 |
| Refbp2 | 30846460 | SmaI | T7 |
| Fox1 | 6821627 | AscI | T3 |
| Hist1h1a | 40126139 | NotI | T3 |
| Med8 | 4022630 | KpnI | T7 |
| Pou6f2 | 6435959 | HpaI | Sp6 |
| Tcfap4 | 6808430 | AscI | T3 |
| Hey2 | N.A. |  |  |
