# Supplementary material for: Homologs of genes expressed in Caenorhabditis elegans GABAergic neurons are also found in the developing mouse forebrain
Source: Neural Dev. 2010 Dec 1;5:32. doi: 10.1186/1749-8104-5-32 (PMC3006369; doi:10.1186/1749-8104-5-32)
Supplement: Additional file 2 — Table S2. Primers used for the generation of subclones from IMAGE clones used to generate in situ hybridization probes in this study. [file 1749-8104-5-32-S2.DOCX]

Table S2. Subclones were produced by standard methods. Specific primers were used to amplify portions of the IMAGE clone templates and the resulting amplicons were subcloned into the pSTBlue-1 vector (Acceptor Vector Kit, Novagen, EMD Chemicals Inc, Merck KGaA, Darmstadt, Germany).

The Nkx2-3 subclone was generated from a BamHI fragment of IMAGE clone 6807512 and subcloned into a pSPT18 vector.

| **Mouse Name** | **IMAGE clone** | **subclone I.D.** | **5' primer** | **3' primer** | **size of insert** | **vector** | **linearizing enzyme** | **anti-sense promoter** |
| --- | --- | --- | --- | --- | --- | --- | --- | --- |
| Foxa1 | 5720113* | 104d | cccctttctccctttcactc | gtgtggagaggcatccttgt | 488 | pSTBlue-1 | BamHI | Sp6 |
| Foxi2 | 30434074* | 221a | tgggtttgccttacttgacc | cccatctgtcctggcatagt | 353 | pSTBlue-1 | HindIII | T7 |
| Ip6k2 | 2609803* | 218a | tggagcgacaggaatcctac | gaggccaacacctagccata | 422 | pSTBlue-1 | HindIII | T7 |
| Arx | 5707995* | 209d | ggcgtctcgttcttgttctc | tgagcgtgacacttctccac | 415 | pSTBlue-1 | BamHI | Sp6 |
| Alx4 | 6506755* | 210a | ctcctgcaggtggtattggt | acctcaagggtggttctgtg | 772 | pSTBlue-1 | BamHI | Sp6 |
| Phox2b | 30360139* | 211a | gccatccagaaccttttcaa | tgctagctcttccctggtgt | 415 | pSTBlue-1 | HindIII | T7 |
| Pax7 | 6843799* | 214a | cagacaaaattgctgctcca | ggtgtcttgtcggttcaggt | 647 | pSTBlue-1 | BamHI | Sp6 |
| Nkx2-3 | 6807512* | 16a1 | N.A. subcloned with 727bp BamHI fragment of IMAGE clone | | 727 | pSPT18 | SalI | T7 |
| C130039O16Rik | 552340* | 222a | ggcgctgaataagctgctac | tccttcctgggttctgtcac | 411 | pSTBlue-1 | SalI | T7 |
| Mier1 | 1395455* | 219b | gactcttttgcccaacgtg | ggaaatgggaggaaggacat | 315 | pSTBlue-1 | HindIII | T7 |
| Rcor1 | 3419361* | 220a | ggcccacagtctggtaagag | gccatcattgaggtgtagca | 409 | pSTBlue-1 | SalI | T7 |
| Foxj3 | 6314981* | 106f10 | cttcccaacagtcccacact | ttaacactgctggcaattcg | 460 | pSTBlue-1 | BamHI | Sp6 |
| Myst3 | 5360083* | 213a | tggagactgcgaggaaaagt | atctgcgtcgtctgactcct | 531 | pSTBlue-1 | HindIII | T7 |
| Myh8 | 1480571* | 217a | gaacagaaacgcaatgctga | aaacccagagaggcaagtga | 419 | pSTBlue-1 | SalI | T7 |
| Clip1 | 3986143* | 216a | aacgagtccctgagaagcaa | cgagctccagtttaccttcg | 568 | pSTBlue-1 | HindIII | T7 |
| Sptbn1 | 6758850* | 30d | aacacagagccctttggaga | gcctcggactctaagcattg | 466 | pSTBlue-1 | SalI | T7 |
| Ncl | 3495665* | 107g3 | acaccagccaaagtcattcc | tcctcctcagccacactctt | 434 | pSTBlue-1 | HindIII | T7 |
| Pnn | 1532266* | 212a | accgacgaatatttggcttg | tgcaaattcgatgcgtctac | 401 | pSTBlue-1 | Sal1 | T7 |
| Foxa3 | 5101155* | 23d | cctccttcgtccacacctta | aaacgcatctgccttcctaa | 426 | pSTBlue-1 | BamHI | Sp6 |
