# Supplementary material for: Homologs of genes expressed in Caenorhabditis elegans GABAergic neurons are also found in the developing mouse forebrain
Source: Neural Dev. 2010 Dec 1;5:32. doi: 10.1186/1749-8104-5-32 (PMC3006369; doi:10.1186/1749-8104-5-32)
Supplement: Additional file 3 — Table S3. Modified Allen Brain Atlas in situ hybridization protocol and Eurexpress II SOP on Tecan Evo GenePaint System. [file 1749-8104-5-32-S3.DOCX]

Table S3. Modified Allan Brain Atlas In situ hybridization protocol and Eurexpress II SOP on Tecan Evo GenePaint System.

| **Step** | **Cycles** | **Time (minutes)** | **Temp °C** |
| --- | --- | --- | --- |
| 3%H2O2 in MeOH | 5 | 5 | 25 |
| PBS | 7 | 5 | 25 |
| 0.2M HCl | 2 | 5 | 25 |
| PBS | 4 | 5 | 25 |
| PK buffer | 1 | 5 | 25 |
| Proteinase K | 2 | 10 | 25 |
| PBS | 7 | 5 | 25 |
| 4% PFA | 2 | 10 | 25 |
| PBS | 7 | 5 | 25 |
| Hyb Solution | 2 | 15 | 25 |
| Ramp-up | 1 | 15 | 63.5 |
| DIG probe hybridization | 1 | 120 | 63.5 |
| DIG probe hybridization | 1 | 210 | 63.5 |
| 5x SSC | 5 | 5 | 63.5 |
| Formamide I | 5 | 10 | 63.5 |
| Formamide II | 5 | 12 | 63.5 |
| 0.1x SSC | 3 | 8 | 63.5 |
| 0.1x SSC | 1 | 8 | 25 |
| NTE | 4 | 5 | 25 |
| Iodoacetamide | 6 | 5 | 25 |
| NTE | 4 | 5 | 25 |
| TNT | 2 | 5 | 25 |
| 4% Sheep Serum | 6 | 5 | 25 |
| TNT | 4 | 5 | 25 |
| TNB blocking buffer | 2 | 10 | 25 |
| TNT | 2 | 5 | 25 |
| Maleate Wash Buffer | 2 | 5 | 25 |
| Blocking reagent | 2 | 10 | 25 |
| Maleate wash buffer | 2 | 5 | 25 |
| TNT | 2 | 5 | 25 |
| NTMT | 3 | 5 | 25 |
| TNT | 4 | 5 | 25 |
| TNB Blocking buffer | 4 | 10 | 25 |
| Anti-DIG POD | 2 | 30 | 25 |
| TNT | 6 | 5 | 25 |
| Tyramide-biotin | 1 | 25 | 25 |
| Maleate wash buffer | 6 | 5 | 25 |
| Neutravidin | 2 | 20 | 25 |
| Maleate wash buffer | 6 | 5 | 25 |
| TNT | 4 | 5 | 25 |
| NTMT | 2 | 5 | 25 |
