# Supplementary material for: Homologs of genes expressed in Caenorhabditis elegans GABAergic neurons are also found in the developing mouse forebrain
Source: Neural Dev. 2010 Dec 1;5:32. doi: 10.1186/1749-8104-5-32 (PMC3006369; doi:10.1186/1749-8104-5-32)
Supplement: Additional file 4 — Table S4. Buffer compositions for in situ hybridization protocol. [file 1749-8104-5-32-S4.DOCX]

Table S4. Buffer compositions for in situ hybridization protocol.

| **Buffer** | **Components** |
| --- | --- |
| 3%H2O2 in MeOH |  |
| PBS | standard recipe with 0.0005% Tween-20 |
| 0.2M HCl | standard recipe with 0.0005% Tween-20 |
| PK buffer | 0.005M EDTA, 0.05M Tris, 0.0005% Tween 20 |
| Proteinase K | PK buffer, 1.5 ug/mL Proteinase K |
| 4% Formaldehyde | 4% Paraformaldehyde by weight dissolved in alkaline water at 60°C, 0.156M NaH2PO4, 0.107M NaOH, pH 7.12 with HCl ,0.0005% Tween-20 |
| Hyb Solution | Ambion B8807g with 1.5 mg/mL dithiothreitol |
| DIG probe hybridization | Ambion B8807g with 0.5 ng/uL DIG-labeled probe |
| 5x SSC | standard recipe with 0.0005% Tween-20 |
| Formamide I | 50% formamide, 2x SSC, 0.0005% Tween-20 |
| Formamide II | 50% formamide, 1x SSC, 0.0005% Tween-20 |
| 0.1x SSC | standard recipe with 0.0005% Tween-20 |
| 1x NTE | 0.5M NaCl, 0.01M Tris (Hydroxymethyl)aminomethane, 0.005M EDTA, 0.0005% Tween-20 |
| Iodoacetamide | 20mM iodoacetamide in 1xNTE |
| 1x TNT | 0.1M Tris (Hydroxymethyl)aminomethane, 0.15M NaCl, 0.00075% Tween-20 |
| 4% Sheep Serum | 4% sheep serum in 1xTNT |
| TNB blocking buffer | 0.5% blocking reagent (Perkin Elmer FP1012), 0.1M Tris (Hydroxymethyl)aminomethane, 0.15M NaCl, 0.0005% Tween-20 |
| Anti-digoxigenin POD | 1:500 Roche anti-DIG (1207733) in 1x TNB |
| Maleate Wash Buffer (MWB) | 0.09M Maleic acid, 0.1M NaCl, 0.2M NaOH, 0.0005% Tween-20, pH 7.5 |
| 1%BR | 1.0% blocking reagent (Roche 1096176) in MWB |
| Tyramide-biotin | 1:100 Tyramide-biotin in TSA buffer (Perkin-Elmer Sat 700) with 0.0005% Tween-20 |
| Neutravidin-AP | 1.2 ug/mL neutravidin-AP (Pierce 31002) in 1%BR |
| NTMT | 0.1M Tris (Hydroxymethyl)aminomethane, 0.05M MgCl, 0.5M NaCl, 0.0005% levamisole, 0.0001% Tween-20 |
| BCIP/NBT | 0.4% NBT (Roche 1383213), 0.3% BCIP (Roche 1383221) in NTMT |
