# Supplementary material for: Homologs of genes expressed in Caenorhabditis elegans GABAergic neurons are also found in the developing mouse forebrain
Source: Neural Dev. 2010 Dec 1;5:32. doi: 10.1186/1749-8104-5-32 (PMC3006369; doi:10.1186/1749-8104-5-32)
Supplement: Additional file 5 — Table S5. Chromosomal position of human homologs of all study genes. [file 1749-8104-5-32-S5.DOCX]

Table S5. Chromosomal position of human homologs of all study genes.

| **Human gene** | **Chromosomal position** |
| --- | --- |
| *MIER1* | 1p31.2 |
| *MED8* | 1p34.1 |
| *FOXJ3* | 1p34.2 |
| *FOXD2* | 1p34-p32 |
| *PAX7* | 1p36.2-p36.12 |
| *SPTBN1* | 2p21 |
| *NCL* | 2q12-qter |
| *IP6K2* | 3p21 |
| *IP6K1* | 3p21.31 |
| *CTNNB1* | 3p22-p21.3 |
| *PHOX2B* | 4p12 |
| *DSPP* | 4q21.3 |
| *PITX2* | 4q25-q26 |
| *PITX1* | 5q31 |
| *NKX2-5* | 5q34 |
| *IP6K3* | 6p21 |
| *TAF11* | 6p21 |
| *TRERF1* | 6p21.1-12.1 |
| *RXRB* | 6p21.3 |
| *HIST1H1A* | 6p21.3 |
| *FOXQ1* | 6p25 |
| *HEY2* | 6q21 |
| *POU6F2* | 7p14-p13 |
| *CUX1* | 7q22.1 |
| *EZH2* | 7q35-q36 |
| *MYST3* | 8p11 |
| *HNF4G* | 8q21 |
| *FOXB2* | 9q21.2 |
| *IPMK* | 10q21 |
| *NKX2-3* | 10q24.2 |
| *PITX3* | 10q25 |
| *FOXI2* | 10q26 |
| *ALX4* | 11p11.2 |
| *PHOX2A* | 11q13.3-q13.4 |
| *RARG* | 12q13 |
| *CUX2* | 12q24.12 |
| *CLIP1* | 12q24.3 |
| *RPGRIP1* | 14q11 |
| *FOXA1* | 14q12-13 |
| *NKX2-1* | 14q13 |
| *PNN* | 14q21.1 |
| *C14orf43* | 14q24.1 |
| *MED6* | 14q24.1 |
| *RCOR1* | 14q32.33 |
| *FOXB1* | 15q21-26 |
| *TFAP4* | 16p13 |
| *MYH11* | 16p13.13-p13.12 |
| *A2BP1* | 16p13.3 |
| *FOXL1* | 16q24 |
| *NCOR1* | 17p11.2 |
| *MYH10* | 17p13 |
| *MYH8* | 17p13.1 |
| *JUP* | 17q21 |
| *EZH1* | 17q21.1-q21.3 |
| *THOC4* | 17q25.3 |
| *FOXA3* | 19q13.2-q13.4 |
| *ZNF541* | 19q13.33 |
| *NKX2-4* | 20p11 |
| *HNF4A* | 20q12-q13.1 |
| *SUV39H1* | Xp11.23 |
| *ARX* | Xp22.13 |
